# Case Report: Expanding the Digenic Variants Involved in Thyroid Hormone Synthesis−10 New Cases of Congenital Hypothyroidism and a Literature Review

**DOI:** 10.3389/fgene.2021.694683

**Published:** 2021-08-12

**Authors:** Rulai Yang, Yijun Lu, Chenxi Yang, Xiaoyu Wu, Junqi Feng, Ling Zhu, Qiang Shu, Pingping Jiang

**Affiliations:** ^1^The Children's Hospitals, Zhejiang University School of Medicine, National Clinical Research Center for Child Health, Hangzhou, China; ^2^Institute of Genetics and Department of Human Genetics, Zhejiang University School of Medicine, Hangzhou, China; ^3^Zhejiang Provincial Key Laboratory of Genetic and Developmental Disorders, Hangzhou, China

**Keywords:** digenic variants, thyroid hormone synthesis, congenital hypothyroidism, genetic counseling, oligogenic cases

## Abstract

Congenital hypothyroidism (CH) is the most common neonatal metabolic disorder. Although it has been understood to be a monogenic disease, some CH patients are reported to carry two or more variants at different genes. Here, ten permanent congenital hypothyroidism (PCH) patients were retrospectively reviewed, with elevated levels of serum thyroid-stimulating hormone and levothyroxine dependence during follow-up between 2015 and 2019. Each affected individual carried digenic variants, which were heterozygous at two of pathogenic genes. In total, five pathogenic genes, *TSHR, TG, TPO, DUOX2* and *DUOXA2*, were simultaneously identified in subjects that were involved in the same metabolic pathway: thyroid hormone biosynthesis. There were digenic variants at TSHR and DUOX2 combined in three patients, *DUOX2* and *TG* combined in two patients, *DUOX2* and *DUOXA2* combined in two patients, *TG* and *DUOXA2* combined in two patients, and *TG* and *TPO* combined in one patient. Additionally, seven novel variants, *TSHR* c.679G>A, *DUOX2* c.127A>T, c.608-619del, c.959T>C, *TG* c.2307G>A, and c.6759_6765del, and *DUOXA2* c.93T>G, were identified in these PCH patients. Along with a literature review on digenic variants in patients with CH, our findings illustrated the complexity of genetic etiology in CH.

## Background

Congenital hypothyroidism (CH) is the most common neonatal metabolic disorder. It has an incidence ranging from 1:1,400 to 1:2,800 live births in many countries (Wassner and Brown, 2015), and it results in severe neurodevelopmental impairment if not treated early and effectively. Primary CH is usually classified into two categories by pathogenesis: thyroid dysgenesis, a defect in thyroid gland development in which a few cases were caused by *FOXE1, NKX2-1, NKX2-5*, and *PAX8*, and thyroid dyshormonogenesis (DH), an intrinsic defect of thyroid hormone biosynthesis caused by *DUOX2, DUOXA2, IYD (DEHAL1), TG, TPO, SLC26A4 (PDS), SLC26A7, SLC5A5 (NIS)*, and *TSHR* (Cangul et al., 2018; Kwak, 2018). Based on the newborn screening (NBS) program and clinical diagnosis, thyroid dyshormonogenes dominate compared to thyroid dysgenesis in the Chinese population upon increased molecular diagnosis (Long et al., 2018; Sun et al., 2018). Whereas thyroid dysgenesis still accounts for more than 69% of primary CH worldwide (Wassner and Brown, 2015; Peters et al., 2018). The inheritance of CH is controversial. Although it has been understood to be autosomal recessive (biallelic) in most cases as a monogenic disorder, a few CH cases appear to be monoallelic in one gene (Nicholas et al., 2016, Fugazzola et al., 2003), or 2 or more variants in different genes (Sriphrapradang et al., 2011; Satoh et al., 2015; Makretskaya et al., 2018; Yamaguchi et al., 2020). Here, we report 10 permanent congenital hypothyroidism (PCH) cases carrying digenic variants in which each affected individual is heterozygous at two of pathogenic genes simultaneously as well as the identification of seven novel genetic variants.

## Case Presentation

During January 2015 and December 2019, the mean incidence of CH was 1:1,093 based on the NBS program in the Children's Hospital, Zhejiang University. CH screening strategies are designed to detect elevated levels of TSH and/or decreased concentrations of thyrocine (T4) (Group for Newborn Screening Society of Child Health Chinese Preventive Medicine Association, 2011). Total 2647 CH cases were diagnosed, of which 148 cases were offered genetic tests, and 66 cases (44.6%) had clear genetic confirmation, either carrying one P/LP variant in a dominate gene or two P/LP variants in a recessive gene. However, another 10 CH patients carrying digenic variants were retrospectively reviewed. They were clinically diagnosed to be PCH with a defect of thyroid hormone biosynthesis based on careful evaluation of clinical features and levothyroxine treatment during follow-up. As shown in Table 1, all patients had initially elevated TSH levels (≥9 μIU/mL), ranging from 9.15 to 25.5 μIU/mL, and were proven to be permanent by receiving a trail off levothyroxine (LT4) at 2–3 years of age. Additionally, the influences of preterm, low-birthweight, and autoimmune thyroid disease on these cases were excluded. The detailed clinical information of the patients was listed in Table 1. With LT4 treatment with a dose of 12.5–33.3 μg per day, all patients had normal ASQ (Ages & Stages Questionnaires) and maintained serum TSH levels ranging from 1 to 10 (mIU/L) with a normal level of free thyroxine (FT4) between 9.01 and 19.05 (pmol/L) cutoff during follow-up. Cases #5 has a goiter by ultrasound during NBS with dimensions 2.3 × 0.9 × 0.8 cm (Right) and 2.2 × 1.0 × 0.8 cm (Left) as previously reported (Wang et al., 2014). There was no compensatory goiter recorded in Case #5 after 1 year with the LT4 supplement.

**Table 1:** The clinical data of 10 primary congenital hypothyroidism cases.

**Cases#**	**Initial TSH**	**Latest record of serum assay**					**Treatments**
**Ages^**†**^**	**(>9 μIU/ml)**	**TSH (mIU/L)**	**T3 (nmol/L)**	**T4 (nmol/L)**	**FT3 (nmol/L)**	**FT4 (pmol/L)**	**Levothyroxine (μg/day)**
		**0.35–4.94**	**0.88–2.44**	**62.68–150.8**	**2.63–5.70**	**9.01–19.05**	
1.6 years	9.15	8.61	1.99	87.47	5.62	15.36	25
2.4 years	13.2	3.652	1.88	126.81	5.95	15.62	12.5
3.5 years	11.8	2.9	2.23	107.08	6.46	17.01	16.7
4.6 years	14.3	3.891	1.71	76.08	4.78	13.51	12.5
5.4 years	25.5	3.787	1.85	144.23	5.98	16.73	33.3
6.2 years	15.1	2.662	2.51	136.62	6.8	14.5	12.5
7.3 years	12.2	8.532	2.28	150.34	6.55	16.16	12.5
8.3 years	10.8	2.962	2.08	129.48	6.27	15.14	16.7
9.6 years	13.7	7.827	2.76	96.44	7.32	12.17	12.5
10.4 years	14.1	3.313	2.29	120.27	8.19	16.38	16.7

*TSH, thyroid-stimulating hormone; T3, Triiodothyronine; T4, Thyroxine; F T3, free Triiodothyronine; FT4, free Thyroxine; y, years*.

### Identification of Digenic and Novel Variants

Identification of causative gene *via* whole-exome sequencing (WES) using peripheral blood was performed for 10 patients. The DNA library was prepared by an Agilent SureSelect Inherited Disease Capture Kit and sequenced using an Illumina HiSeq 2500 platform. All sequencing reads were mapped to the human reference genome (GRCh37) by BWA (Li and Durbin, 2010) and annotated by ANNOVAR (http://annovar.openbioinformatics.org). A series of automatic tools (SIFT, Polyphen, MutationTaster, etc.) were used to predict the functional significance of variants (Table 2). DNA samples from family #1, #3, #4, #6, #7, #8, and #10 were verified further by Sanger sequencing (Supplementary Figure 1).

**Table 2:** Genetic variants and their prediction on protein function.

**Case#**	**Gene**	**cDNA and amino acid change**	**ExonicFunc.refGene**	**Resourse**	**ACMG interpretation**	**ACMG classification**	**Allele Frequency (ExAC ALL)**	**Prediction**				
								**SIFT**	**Polyphen2_HDIV**	**LRT**	**Mutation Taster**	**FATHMM**
1	*TSHR*	**c.679G>A (p.G227R)**	Nonsynonymous SNV	Maternal	PM1+PM2+PP3	VUS	–	D	D	D	D	D
	*DUOX2*	**c.127A>T (p.N43Y)**	Nonsynonymous SNV	Paternal	PM2+PP3	VUS	0.09225%0	D	D	D	D	D
2	*TSHR*	c.1574T>C (p.F525S)	Nonsynonymous SNV	Maternal	PM1+PM2+PP3+PP5	LP	0.1%0	T	D	D	D	T
	*DUOX2*	**c.608_619del (p.L203_P207delinsP)**	Nonframeshift deletion	Maternal	PVS1+PM2+PM4	P	–	.	.	.	.	.
3	*TSHR*	c.733G>A (p.G245S)	Nonsynonymous SNV	Maternal	PM2+PP3+PP5	VUS	0.1%0	D	D	D	D	D
	*DUOX2*	c.3516_3531del (p.K1174Sfs^*^12)	Frameshift deletion	Paternal	PVS1+PM2+PP5	P	0.008266%0	.	.	.	.	.
4	*DUOX2*	c.2654G>T (p.R885L)	Nonsynonymous SNV	Maternal	PM2+PP3+PP5	VUS	0.3%0	D	D	D	D	T
	*TG*	c.6759_6765del (p.S2254Mfs^*^88)	Frameshift deletion	Paternal	PVS1+PM2	LP	–	.	.	.	.	.
5	*DUOX2*	c.3516_3531del (p.K1174Sfs^*^12)	Frameshift deletion	Paternal	PVS1+PM2+PP5	P	0.008266%0	.	.	.	.	.
	*TG*	c.2307G>A (p.W769^*^)	Stopgain	*de novo*	PVS1+PS2+PM2	P	–	.	.	D	A	.
6	*DUOX2*	c.4027C>T (p.L1343F)	Nonsynonymous SNV	Maternal	PM1+PM2+PP3+PP5	LP	0.5%0	T	P	D	D	T
	*DUOXA2*	c.738C>G (p.Y246^*^)	Stopgain	Paternal	PVS1+PM2+PP5	P	0.2%0	.	.	N	D	.
7	*DUOX2*	**c.959T>C (p.L320P)**	Nonsynonymous SNV	Maternal	PM2+PP3	VUS	0.03304%0	D	P	N	D	T
	*DUOXA2*	c.738C>G (p.Y246^*^)	Stopgain	Paternal	PVS1+PM2+PP5	P	0.2%0	.	.	N	D	.
8	*TG*	c.3040G>A (p.D1014N)	Nonsynonymous SNV	Paternal	PM1+PM2+PP5	VUS	0.02472%0	T	B	N	N	T
	*DUOXA2*	**c.93T>G (p.F31L)**	Nonsynonymous SNV	Maternal	PM2+PP3	VUS	0.2%0	D	D	D	D	T
9	*TG*	c.3808C>T (p.R1270C)	Nonsynonymous SNV	Maternal	PM2+PP5	VUS	0.2%0	D	D	N	N	T
	*DUOXA2*	c.413dupA (p.Y138^*^)	Stopgain	Maternal	PVS1+PM2+PP5	P	0.2%0	.	.	D	A	.
10	*TG*	c.5791A>G (p.I1931V)	Nonsynonymous SNV	Maternal	PM2+PP5	VUS	0.2%0	T	B	N	N	T
	*TPO*	c.2647C>T (p.P883S)	Nonsynonymous SNV	Paternal	PM2+PP5	VUS	0.5%0	T	B	.	N	T

*The reference gene version is GRCh37/hg19. DUOX2(NM_014080.4), dual oxidase 2; DUOXA2 (NM_207581.4), dual oxidase maturation factor 2; TSHR(NM_000369.2),TSH receptor; TG (NM_003235.4), thyroglobulin; TPO (NM_000547.5), thyroid peroxidase*.

*D, Damaging/Deleterious/disease causing; P, possibly damaging; N, Neutral; A, disease causing automatic; T, Tolerated*.

*Truncating protein as a result of stopgain or deletion is considered pathogenic according to ACMG guidelines. Novel variants are in bold*.

* *stopgain or truncated protein*.

**Figure 1:**
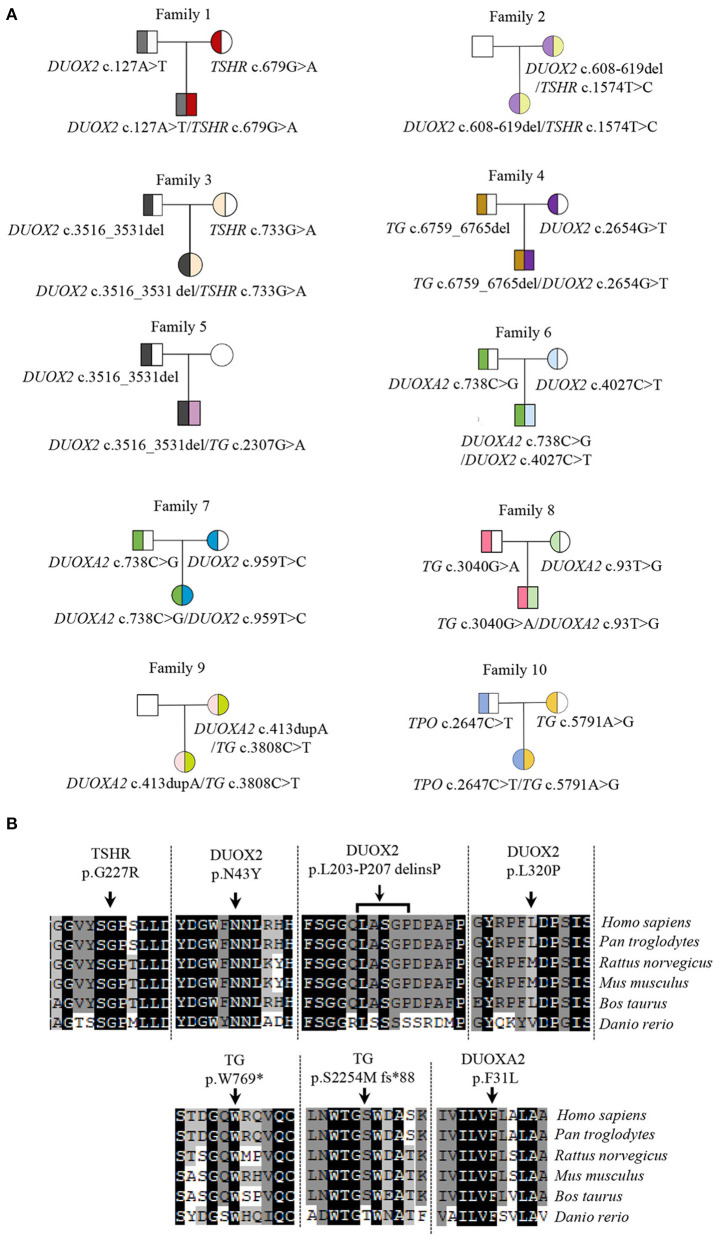
Variants in CH patients and their conservative analysis. **(A)** Genotypes of CH pedigrees; **(B)** Conservative analysis of seven novel variants in species. The reference sequences for *TSHR, DUOX2, TG*, and *DUOXA2* in species are followed as: *TSHR: Homo sapiens, NP_000360.2; Pan troglodytes, XP_009426511.1; Rattus norvegicus, NP_037020.2; Mus musculus, NP_035778.3; Bos taurus, NP_776631.1; Danio rerio, NP_001139235.1. DUOX2: Homo sapiens, NP_054799.4; Pan troglodytes, XP_009427327.1; Rattus norvegicus, NP_077055.2; Mus musculus, NP_001349684.1; Bos taurus, XP_005211958.1; Danio rerio, XP_002666953.2. TG: Homo sapiens, NP_003226.4; Pan troglodytes, XP_016815373.2; Rattus norvegicus, NP_112250.2; Mus musculus, NP_033401.2; Bos taurus, NP_776308.1; Danio rerio, NP_001316794.1. DUOXA2: Homo sapiens, NP_997464.2; Pan troglodytes, XP_001146826.2; Rattus norvegicus, NP_001178894.1; Mus musculus, NP_080053.1; Bos taurus, XP_002690989.1; Danio rerio, XP_017209762.1*.

The majority of dyshormonogenesis has an identifiable genetic basis since there are more than 10 genes reported to be involved in thyroid hormone biosynthesis (Kwak, 2018). All identified genes and variants were summarized in Figure 1. Five causative genes, *TSHR, TG, TPO, DUOX2*, and *DUOXA2*, were identified among the 10 patients. *DUOX2* was detected in seven patients, followed by *TG* in five patients, *DUOXA2* in four patients, *TSHR* in three patients, and *TPO* in one patient. There were digenic variants involving *TSHR* and *DUOX2* in Case #1 (*TSHR* c.679G>A and *DUOX2* c.127A>T), #2 (*TSHR* c.1574T>A and *DUOX2* c.608-619del), and #3 (*TSHR* c.733G>A and *DUOX2* c.3516_3531del), *DUOX2* and *TG* in Case #4 (*DUOX2* c.2654G>T and *TG* c.6759_6765del) and #5 (*DUOX2* c.3516_3531del and *TG* c.2307G>A), *DUOX2* and *DUOXA2* in Case #6 (*DUOX2* c.4027C>T and *DUOXA2* c.738C>G) and #7 (*DUOX2* c.959T>C and *DUOXA2* c.738C>G), *TG* and *DUOXA2* in Case #8 (*TG* c.3040G>A and *DUOXA2* c.93T>G) and #9 (*TG* c.3808C>T and *DUOXA2* c.413dupA), and *TG* and *TPO* in Case #10 (*TG* c.5791A>G and *TPO* c.2647C>T). In total, 18 variants were identified: 12 missense, 2 nonsense, and 4 frameshifts. These resulted from three deletions and one duplication. Five truncating proteins were observed in 7 cases, including *DUOX2* p.K1174S fs^*^12 (c.3516_3531del) in Cases #3 and #5, *TG* p.S2254M fs^*^88 (c.6759_6765del) in Case #4, p.W769^*^ (c.2307G>A) in Case #5, *DUOXA2* p.Y246^*^ (c.738C>G) in Cases #6 and #7, and p.Y138^*^ (c.413dupA) in Case #9. Usually, truncating variants were pathogenic based on American College of Medical Genetics (ACMG) guidelines. Variants were mostly transmitted from both parents, although two heterozygous variants of Case #2 and #9 were solely from the mother (Figure 1). Moreover, a *de novo* variant resulting in a truncated protein, *TG* c.2307G>A (p. W769^*^), was detected in Case #5 (Supplementary Figure 1). Among these 18 variants, 7 were novel, identified as *TSHR* c.679G>A (p.G227R); *DUOX2* c.127A>T (p.N43Y), c.608-619del (p.L203-P207delinsP) and c.959T>C (p.L320P); *TG* c.2307G>A (p.W769^*^) and c.6759_6765del (p.S2254Mfs^*^88); and *DUOXA2* c.93T>G (p.F31L). All the novel variants were localized in highly conserved regions of each protein (Figure 1) and predicted to be potential pathogenic variants by functional consequences annotation through multiple software (Table 2).

## Discussion

Most cases of CH are common endocrine disorders caused by biallelic or monoallelic variants in one gene. With the widespread use of newborn screening programs and the application of genetic testing, some cases were found to carry two or more variants at different genes (Satoh et al., 2015; Nicholas et al., 2016; Sun et al., 2018; Yamaguchi et al., 2020), indicating the complexity of genetic etiology in CH. Cases with two or more variants in different genes were usually understood to be oligogenic cases, compared to those in biallelic and monoallelic cases (Yamaguchi et al., 2020). Here, we present 10 PCH cases carrying digenic variants in genes involved in thyroid hormone biosynthesis. Similar to our findings, another 58 cases harboring digenic variants were reported elsewhere (Supplementary Table 1). A total of 24 cases had digenic variants in *TSHR* and *DUOX2*, including 5 cases out of 220 Chinese CH (Fang et al., 2019) and 6 cases in Japanese patients (Abe et al., 2018; Yamaguchi et al., 2020). The coexistence of heterozygous variants in *TSHR* and *DUOX2* was also revealed in Caucasian cases (Makretskaya et al., 2018; Sasivari et al., 2019). More digenic variants were heterozygous in two causative genes, including combined *DUOX2* and *TG* in 13 patients (Löf et al., 2016; Fan et al., 2017; Long et al., 2018; Sun et al., 2018; Yamaguchi et al., 2020), *DUOX2* and *DUOXA2* in 4 patients (Zheng et al., 2016; Yamaguchi et al., 2020), *DUOX2* and *TPO* in 3 patients (Matsuo et al., 2016; Long et al., 2018; Makretskaya et al., 2018), *TG* and *TPO* in 6 patients (Nicholas et al., 2016; Makretskaya et al., 2018; Yamaguchi et al., 2020), and *TG* and *SLC26A4* in 2 patients (Löf et al., 2016; Sun et al., 2018). Moreover, it was also demonstrated that 23% of Italian CH patients harbored pathogenic variants in more than one gene (Filippis et al., 2017), indicating that there was no ethnicity limiting the digenic form but rather a frequency of dyshormonogenesis-associated variants. As shown in Supplementary Table 1, eight genes (*TSHR, TG, DUOX2, DUOX1, DUOXA2, TPO, IYD*, and *SLC26A4*) were present in those oligogenic cases. The higher frequency genes were *DUOX2* (35.3%), *TSHR* (22.8%), and *TG* (22.8%). This was consistent with prior studies showing that *DUOX2* and *TSHR* variants were more prevalent in Chinese, Japanese, and Korean patients (Jin et al., 2014; Fu et al., 2016; Fang et al., 2019; Yamaguchi et al., 2020). Higher frequent TG variants were detected in the Sudanese population (Bruellman et al., 2020). However, only two cases harbored variants of *IYD* that one individual combined with *TG* (Makretskaya et al., 2018) and the other one with *DUOX1*(Sun et al., 2018). The digenic variants thereby seemed to be common in CH, but it is somewhat challenged in the variant interpretation by the dominant effect of some of these variants. For example, there were monoallelic variants reported in *DUOX2* (Moreno et al., 2002), and later this turned out to be associated with transient hypothyroidism (Wang et al., 2014; Matsuo et al., 2016).

**Figure 2:**
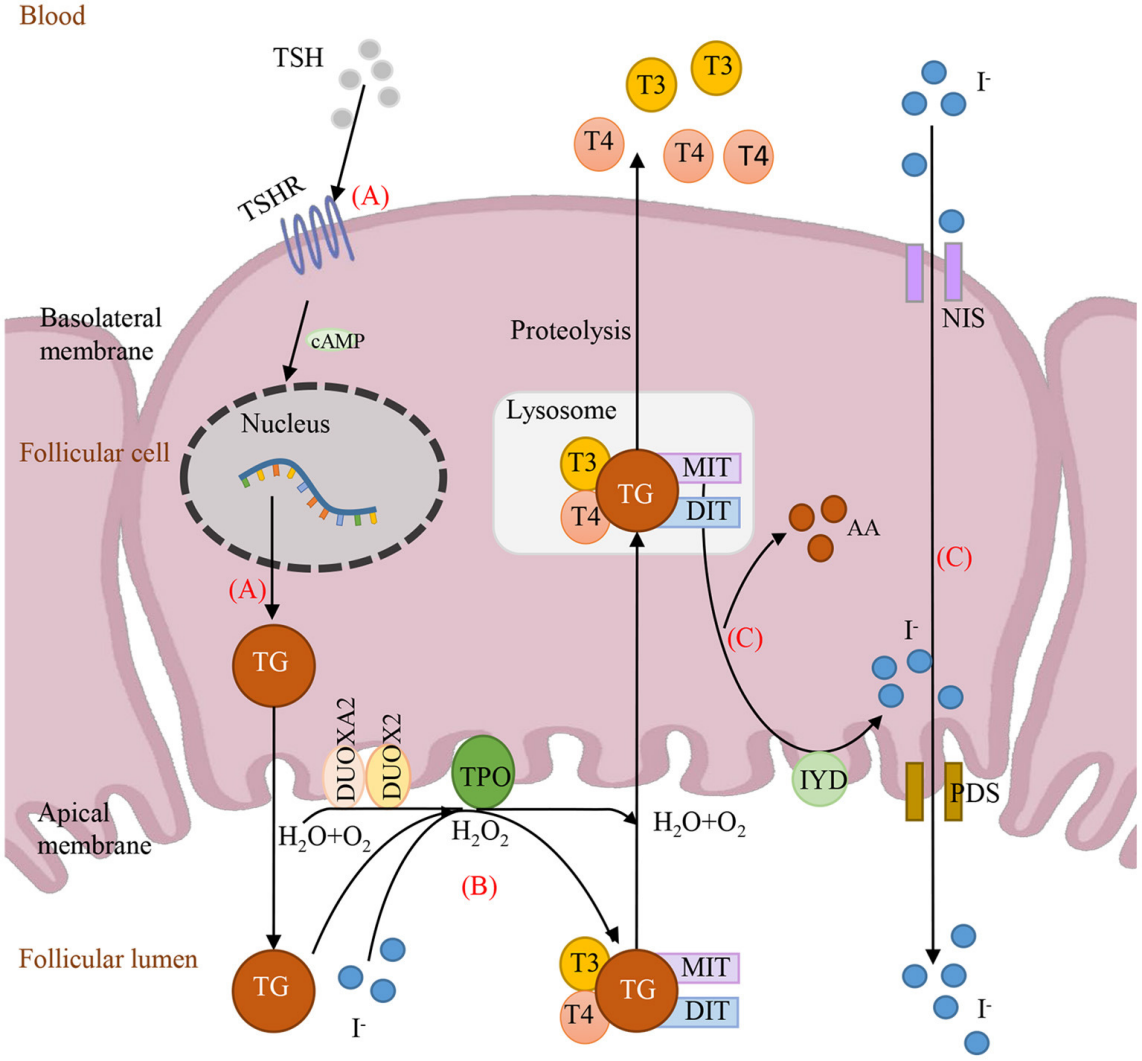
Schematic depiction of the causative genes involving in thyroid hormone synthesis. (A) TSH binds to TSHR and then activates TG expression. (B) The iodide organification, with substrates TG and H_2_O_2_, which is completed by enzymes TPO, DUOX2 and DUOXA2. (C) Iodide recycled by IYD and transported by NIS and PDS. TSH, thyroid-stimulating hormone; T3, Triiodothyronine; T4, Thyroxine; MIT, monoiodotyrosine; DIT, diiodotyrosine; DUOX2, dual oxidase 2; DUOXA2, dual oxidase maturation factor 2; TSHR, TSH receptor; TG, thyroglobulin; TPO, thyroid peroxidase; AA, amino acid.

To date, all reported genes with digenic variants are involved in the same metabolic pathway: thyroid hormone biosynthesis. As shown in Figure 2, the thyroid hormone is synthesized at the apical surface of polarized thyroid follicular cells, where the initial step is the binding of TSH to its receptors (TSHR) in the basolateral membrane, activating TG expression. To date, only 5 oligogenic cases carried heterozygous *TSHR* and *TG* variants in the Chinese and Japanese population (Fu et al., 2016; Yamaguchi et al., 2020). However, most cases of heterozygous *TSHR* (77.4%, 24/31) were combined with heterozygous *DUOX2* as shown in Supplementary Table 1 (involved in steps 1 and 2). Subsequently, TG, TPO, and the DUOXs (DUOX2 and DUOX1) and their accessory protein DUOXA2 are involved in iodide oxidation to form T4 and T3 (Kwak, 2018). Here, 7 out of 10 cases were caused by two of the five genes in this step. Additionally, overall 32 oligogenic cases (47%, 32/68) carried the combination of two heterozygous variants in two genes in this step, indicating that iodide organification defects may be more common in CH patients. Genes, *IYD/DEHAL1, NIS/SLC5A5* and PDS/SLC26A4 involved in recycling of T4, T3, iodide and tyrosine (Spitzweg et al., 2000). Limited by the number of cases, there was no variant detected in *NIS* as elsewhere (Long et al., 2018). Only a few variants have been reported to date in *IYD* and *PDS*. As shown in Supplementary Table 1, there were only three cases carrying heterozygous *PDS* and two cases carrying heterozygous *IYD*. Theoretically, any defects of these eight proteins in substrates, enzymes, and transport molecules in the same metabolic pathway led to thyroid dyshormonogenesis. In fact, our data and recent evidence revealed that the combinations of two pathogenic genes predominantly happened in iodide organification and then in TG expression.

The limitation here is the lack of a parental phenotype, and all our cases are simplex cases. Especially in Case#2 and #9, the parental phenotype is critical in understanding the functional effects of digenic variants. Unfortunately, the mothers carrying the same two variants refused to test their TSH levels. Moreover, the digenic variants in Case#1, #8, and #10 were classified to be VUS according to the ACMG guidelines, which need more cases or further functional experiments to evaluate their damage prediction.

Summarily, we reported here 10 PCH cases with digenic variants involved in the same metabolic pathway: thyroid hormone biosynthesis. To date, 68 CH patients have been reported harboring digenic variants in this metabolic pathway, including genes *TSHR, TG, DUOX2, DUOX1, DUOXA2, TPO, IYD*, and *SLC26A4* with a high frequency of *DUOX2, TSHR*, and *TG*. The data present here will extend our awareness of the complexity of genetic etiology in CH.

## Data Availability Statement

The original contributions presented in the study are publicly available in NCBI using accession number PRJNA734721.

## Ethics Statement

The studies involving human participants were reviewed and approved by the Research Ethics Committees, Children's Hospital of Zhejiang University School of Medicine. Written informed consent to participate in this study was provided by the participants' legal guardian/next of kin. Written informed consent was obtained from the individual(s), and minor(s)' legal guardian/next of kin, for the publication of any potentially identifiable images or data included in this article.

## Author Contributions

PJ and QS performed the conception, analysis, and interpretation of data. RY and LZ recruited patients and performed clinical evaluation. YL, CY, XW, and JF conducted the mutational sequencing and data analysis. PJ, YL, RY, and QS drafted and revised the article. All authors contributed to the article and approved the submitted version.

## Conflict of Interest

The authors declare that the research was conducted in the absence of any commercial or financial relationships that could be construed as a potential conflict of interest.

## Publisher's Note

All claims expressed in this article are solely those of the authors and do not necessarily represent those of their affiliated organizations, or those of the publisher, the editors and the reviewers. Any product that may be evaluated in this article, or claim that may be made by its manufacturer, is not guaranteed or endorsed by the publisher.

## Abbreviations

CH: congenital hypothyroidism
PCH: permanent congenital hypothyroidism
TSH: thyroid-stimulating hormone
TSHR: thyroid-stimulating hormone receptor
T3: triiodothyronine
T4: thyroxine
MIT: monoiodotyrosine
DIT: diiodotyrosine
DUOX2: dual oxidase 2
DUOX1: dual oxidase 1
DUOXA2: dual oxidase maturation factor 2
TG: thyroglobulin
TPO: thyroid peroxidase
SLC26A4 (PDS): solute carrier family 26 member 4
SLC26A7: solute carrier family 26 member 7
SLC5A5 (NIS): solute carrier family 5 member 5 (sodium iodide symporter).
