# Supplementary material for: Case Report: Expanding the Digenic Variants Involved in Thyroid Hormone Synthesis−10 New Cases of Congenital Hypothyroidism and a Literature Review
Source: Front Genet. 2021 Aug 12;12:694683. doi: 10.3389/fgene.2021.694683 (PMC8397485; doi:10.3389/fgene.2021.694683)
Supplement: Supplementary file 1 [file Data_Sheet_1.PDF]

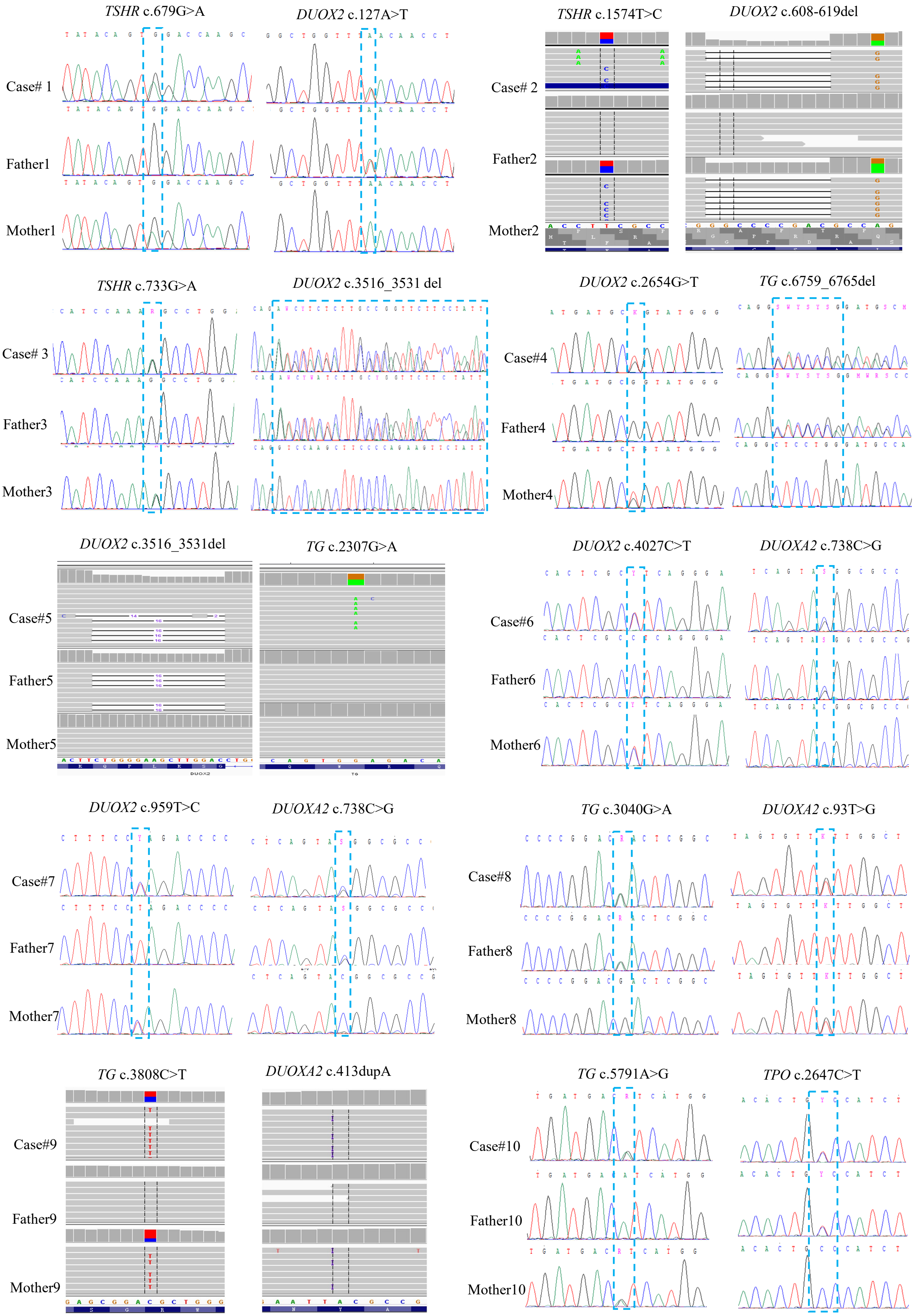

Supplementary Figure 1. Verification of variants by Sanger sequencing or next generation sequencing
