# Supplementary material for: Case Report: Expanding the Digenic Variants Involved in Thyroid Hormone Synthesis−10 New Cases of Congenital Hypothyroidism and a Literature Review
Source: Front Genet. 2021 Aug 12;12:694683. doi: 10.3389/fgene.2021.694683 (PMC8397485; doi:10.3389/fgene.2021.694683)
Supplement: Supplementary file 2 [file Data_Sheet_2.PDF]

Supplementary Table 1. Sixty-eight CH cases harboring digenic variants and the distribution of genes

Cases	TSHR	TG	DUOX2	TPO	DUOXA2	IYD	SLC26A4	DUOX1	Reference
1	p.R450H		p.E327*						
2/3†	p.R450H		p.K530*						Abe,et al.,2018
4	p.R450H		p.V779M						
5	p.R109Q		p.K530*						Fan,et al.,2017
6		p.P1012L	p.E879K						
7	p.R109Q		p.R1211H						
8	p.V354I		p.R885L						
9	p.K751R		p.N476K						Fang,et al.,2019
10	p.A204V		p.G1513R						
11	p.M164K		p.R885Q						
12	p.Y613C		p.K530*						
13	p.R109Q		p.K530*						Fu,et al.,2016
14	p.696C		p.E879K						
15	p.A526T	IVS3 + 2T > G							
16	p.G132R		p.H678R						Jin,et al.,2014
17	p.R450H		p.A1123T						
18		p.P118L	p.P303R						Löf,et al.,2016
19		p.S1139L					p.L597S		
20	p.G245S		p.S965fs*30						
21		p.R2185W		p.P70A					
22		p.R152H		p.S97*					
23		p.P1494L		p.V484M					Makretskaya,et al.,2018
24			p.R1535G	p.T321I					
25			p.S965fs*30				p.M147I		
26		p.A993T				p.T273M			
27		p.Q1644E		p.R491H					Nicholas,et al.,2016
28	p.C41S		p.Q202Tfs						Sasivari,et al.,2019
29	p.R450H		p.K628Rfs*11						
30	p.R310H		p.G488R						
31	p.R450H				p.V154M				
32	p.G245S	p.P695S							
33	p.R450H	p.K2733M							
34/35†		p.R1066H	p.G488R						
36		p.R1998*	p.S965L						Yamaguchi,et al.,2020
37		p.R1066H	p.A1060V						
38		p.V129I		p.R367*					
39		p.P1029Pfs*2	p.E973K						
40		p.Q830E	p.V779M						
41		p.P777L			p.Y138*				
42			p.K628Rfs*11		p.S37R				
43			p.E879K					p.R139S	
44			p.R885Q		c.554+5C>T				Zheng,et al.,2016

45	p.G227R		p.N43Y						
46	p.F525S		p.L203_P207delinsP						
47	p.G245A		p.K117Sfs*12						
48		p.S2254Mfs*88	p.R885L						
49		p.W769*	p.K117Sfs*12						This study
50			p.L1343F		p.Y246*				
51			p.L320P		p.Y246*				
52		p.D1014N			p.F31L				
53		p.R1270C			p.Y138*				
54		p.I1931V		p.P883S					
55		p.I444V	p.M1132K						
56	p.R109Q		p.R1211H						Sun,et al.,2018
57			p.G624Afs*15					p.T1079N	
58		p.R1829P					p.I363L		
59						p.M236V		p.R50Q	
60		p.T728K	p.R1211C						
61									
62									
63									
64									Long,et al.,2018
65									
66									
67									
68									Matsuo,et al.2016
Sum	31	31	48	10	8	2	3	3	136

The reference gene version is GRCh37/hg19. *DUOX2*(NM_014080.4), dual oxidase 2; *DUOXA2* (NM_207581.4),dual oxidase maturation factor 2; *TSHR*(NM_000369.2),TSH receptor; *TG* (NM_003235.4), thyroglobulin ; *TPO* (NM_000547.5), thyroid peroxidase; *IYD* (NM_203395), iodotyrosine deiodinase; *SLC26A4 (*PDS) (NM_000441), solute carrier family 26 member 4; *DUOX1* (NM_017434.5), dual oxidase 1

†, two cases with the same genotype
